# Emergence of *Leuconostoc mesenteroides* as a causative agent of oozing in carrots stored under non‐ventilated conditions

**DOI:** 10.1111/1751-7915.12753

**Published:** 2017-08-22

**Authors:** Yael Lampert, Barak Dror, Noa Sela, Paula Teper‐Bamnolker, Avinoam Daus, Shlomo Sela (Saldinger), Dani Eshel

**Affiliations:** ^1^ Department of Postharvest and Food Sciences ARO The Volcani Center Rishon LeZion Israel; ^2^ Department of Food Quality and Safety ARO The Volcani Center Rishon LeZion Israel; ^3^ Department of Plant Pathology and Microbiology The Robert H. Smith Faculty of Agriculture, Food and Environment The Hebrew University of Jerusalem Rehovot Israel; ^4^ Department of Plant Pathology and Weed Science ARO The Volcani Center Rishon LeZion Israel

## Abstract

Long‐term storage and transport of post‐harvest carrots (*Daucus carota* L.) require a low‐temperature, high‐relative‐humidity environment, usually with low ventilation. Following long‐term storage, a slimy exudate (oozing) often appears on the carrots, leading to severe spoilage. We characterized the environmental conditions leading to these symptoms and identified the causative agent. Simulation of non‐ventilated storage conditions revealed accumulation of CO_2_ (to 80%) and ethanol (to 1000 ppm); then, a transparent exudate appeared on the carrot surface which, upon ventilation, developed into tissue browning and soft rot. Peels from oozing carrots contained over 10‐fold the total bacterial counts of healthy carrots. The total peel microbiome was determined by 16S rDNA sequencing. During oozing stage, the surface of carrots incubated in a CO_2_‐rich (98%) environment harboured a bacterial population dominated by *Lactobacillales* and *Enterobacteriales*, differing markedly from those incubated in air. Three prevalent bacterial isolates from the oozing carrots were identified as *Pantoea agglomerans*,* Rahnella aquatilis* and *Leuconostoc mesenteroides*. Inoculation of carrot discs with *L. mesenteroides*, but not the others, induced oozing under high CO_2_, suggesting that this bacterium is responsible for oozing of stored carrots. These findings should enable development of approaches to preventing carrot spoilage during long‐term storage.

## Introduction

Carrot (*Daucus carota* L.) is an economically important staple food worldwide. Optimal storage conditions consist of refrigeration at 1°C and 98% relative humidity (Phan *et al*., 1973; Seljåsen *et al*., 2001). These conditions prevent moisture loss from the carrots, help to retain their quality for up to 7 months and enable overseas export (Godfrey and Marshall, 2002). During harvest, transport and processing carrot tissue damage triggers non‐microbial and microbial spoilage during storage and a subsequent negative impact on sensory quality (Martínez‐Hernández *et al*., 2015). Post‐harvest diseases can be triggered, and they are considered as an important limiting factors to long‐term storage of carrot products (Martínez‐Hernández *et al*., 2015). It has been suggested that spoilage development in stored carrots is related to the presence of organic debris or soil on the roots, the degree of wounding from mechanical harvesting and pre‐storage washing, and the length of the storage period (Goodliffe and Heale, 1977; Godfrey and Marshall, 2002). Bacterial soft rot of stored carrots, for instance, caused by the bacterial pathogens, is known to contaminate carrots in the field before harvest and readily spread in the washing water during the post‐harvest handling (Klaiber *et al*., 2005). Another type of rot, induced by carrot brushing, is termed ‘black root rot’ and is caused by two fungi, *Thielaviopsis basicola* and *Chalaripsis thielavioides*. These fungi causing large black superficial patches on the carrot roots infect them through wounds or abrasions (Weber and Tribe, 2004; Eshel *et al*., 2009). Other types of common post‐harvest pathogens of carrots include other fungi, such as *Botrytis cinerea*,* Rhizoctonia carotae* (Geeson *et al*., 1988) and *Sclerotinia sclerotiorum* (Liew and Prange, 1994), and the bacterial pathogens *Pectobacterium (Erwinia) carotovorum* (Michalik *et al*., 1992), *(Erwinia) chrysanthemi* (Farrar *et al*., 2000), *Pseudomonas viridiflava* (Wells *et al*., 1998) and *Pseudomonas marginalis* (Hunter and Cigna, 1981).

A variety of methods has been proposed to overcome post‐harvest carrot diseases in order to minimize losses. These methods are not only applicable for the whole stored carrot, but also for other derived products such as minimally processed, sliced and shredded carrots (Tzortzakis, 2016; Villafañe, 2017). Generally, these approaches can be divided into physical, chemical, biological and combined, based on the tool being used. The physical methods include treatments like low‐dose irradiation or microwaving (Kamat *et al*., 2005; Martínez‐Hernández *et al*., 2015), steam application (Gan‐Mor *et al*., 2011) and modified atmosphere (Larsen and Wold, 2016). The chemical treatments include usually washing with chlorinated water, and final treatment with fungicide (iprodione, etc.) is a widely used method of sanitizing brushed and fresh‐cut carrots, thus reducing their initial microbial loads (Eshel *et al*., 2009). As the potential risks of the latter group are accumulating and safety regulations limit the use of these chemical products, more and more biological applications are entering the market. Among these applications are different kinds of edible chitosan coatings (reviewed by Villafañe, 2017). Lastly combined methods are encouraging the synergetic use of several tools to maximize their efficiency (Eshel *et al*., 2009; Eshel, 2011).

In Israel, carrots usually undergo minimal processing, which includes removal of inedible parts, washing and brushing to remove soil and the outer peel of the root (Eshel *et al*., 2009). Exported carrots are transported overseas in large containers at 4–6°C. In recent years, some shipments from Israel have been rejected due to development of oozing followed by soft rot during shipment, causing high economic losses to the farmers. The aims of this study were to characterize the environmental conditions that may trigger the oozing symptoms in stored carrots and to isolate and identify the causative agent.

## Results

### Non‐ventilated storage conditions induce oozing symptoms

**Figure 1:**
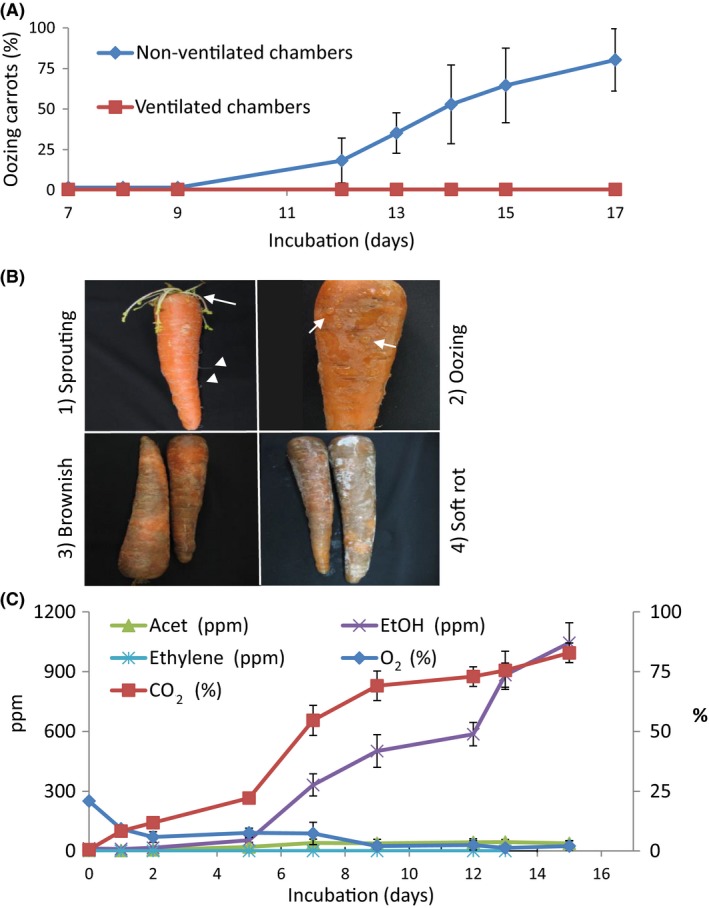
Non‐ventilated storage conditions induce CO_2_ and ethanol volatile accumulation followed by oozing symptoms. A. Percentage of oozing carrots in non‐ventilated chambers as compared to ventilated chambers. B. Typical carrot symptoms in the sequence of appearance: (1) sprouting (arrow) and rooting (arrow‐head) of carrots during storage under ambient air; (2) oozing (arrows) carrots in non‐ventilated chambers after 9–12 days; (3) brownish colour appearing on stored oozing carrots after ventilation for 24 h; (4) soft rot after oozing followed by ventilation for 3 days. C. Changes of volatile levels in non‐ventilated chambers. Error bars, ± SD,* n* = 5. Acet, acetaldehyde; EtOH, ethanol.

To determine the effect of ventilation on symptom development, whole carrots were stored in either sealed (non‐ventilated) or open chambers. After 9–12 days of storage, a transparent exudate (oozing) was observed on the surface of the stored carrots in the non‐ventilated chambers (Fig. 1A, B), and the average percentage of oozing carrots increased sharply to 80% after 17 days in the non‐ventilated chambers (Fig. 1A). In the ventilated chambers, no symptoms were observed (Fig. 1A), but sprouting of leaves were observed in all carrots (Fig. 1B). Oozing carrots became brownish within 24 h after opening the non‐ventilated chambers (Fig. 1B). After further incubation in ambient atmosphere for another 48 h, soft rot developed, with the brownish carrot tissues becoming soft and ‘slimy’ (Fig. 1B). As the oozing phenomenon frequently results from microbial activity (Davis and Nu, 2007; Horst, 2013), it could be assumed that the elevated level of CO_2_ in the non‐ventilated chamber induced specific microbial activity that resulted in the oozing phenotype.

### CO_2_ and ethanol volatiles accumulate under non‐ventilated conditions

To determine the effect of storage conditions on volatile composition and a possible correlation to carrot spoilage, the presence of CO_2_, oxygen, acetaldehyde, ethanol and ethylene was determined in the headspace of the non‐ventilated chambers containing stored carrots for up to 15 days. Accumulation of CO_2_ was observed as early as day 1 of storage, reaching 80% after 15 days (Fig. 1C). A sharp, ca. threefold increase in CO_2_ concentration occurred between days 5 and 9. Ethanol concentration began to increase after 5 days of storage to 1000 ppm on day 15 (Fig. 1C). Conversely, O_2_ concentration decreased gradually until day 9 and remained constant at 2% until day 15. Acetaldehyde concentration remained low throughout the storage period. No ethylene was detected during storage (Fig. 1C).

### CO_2_ induces oozing in carrots

**Figure 2:**
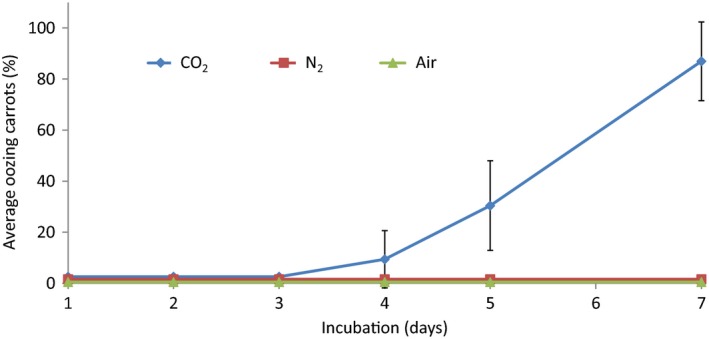
Only CO_2_ flushing causes oozing of stored carrot in non‐ventilated conditions. CO_2_, N_2_ and air were flushed daily into the chambers for 7 days, and then, incubation chambers were ventilated for 24 h. Error bars, ± SD,* n* = 5.

The apparent correlation between CO_2_ accumulation, O_2_ reduction and carrot oozing suggested that anaerobic conditions may induce carrot oozing and spoilage. To determine whether oozing symptoms are caused specifically by CO_2_, non‐ventilated chambers containing carrots were flushed once a day, for 7 days, with CO_2_, N_2_ or air. Only the CO_2_ atmosphere resulted in carrot oozing, starting after 3–4 days of incubation. The percentage of oozing carrots under the high CO_2_ atmosphere reached 86.9 ± 15.4% on day 7 (Fig. 2). The N_2_ or ambient air atmosphere had no visible effect on oozing (Fig. 2). After 7 days, the chambers were opened and the carrots were examined daily for symptom development. Within 24 h, all the carrots that had been stored in 98% CO_2_ atmosphere, as opposed to none of the carrots in 98% N_2_ or air, became brownish. These data suggested that high CO_2_ level, rather than lack of O_2_, induces oozing and the consequent development of brownish colour following air ventilation.

### Oozing symptoms correlate with bacterial count

**Figure 3:**
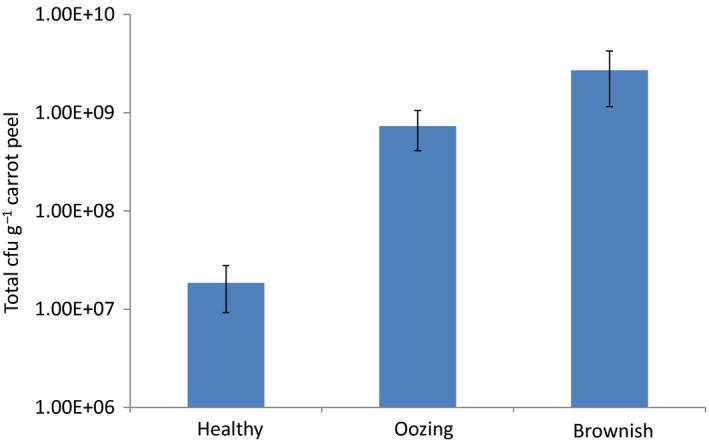
Heterotrophic bacterial counts on carrot peels derived from healthy and symptomatic carrots. Bacteria were plated on PCA and incubated at 28°C for 48 h. Error bars, ± SD,* n* = 5.

To determine the microorganisms associated with the oozing and brownish colour symptoms, peel extracts derived from healthy, oozing and brownish carrots were serially diluted and plated on plate count agar (PCA) and potato dextrose agar (PDA)–chloramphenicol to isolate bacteria and fungi respectively. Fungal isolates were not consistently cultured from oozing and brownish carrot samples (data not shown), suggesting that these symptoms result from the presence of bacterial pathogens. The total count of mesophilic aerobic bacteria on oozing carrots was more than 10‐fold higher [7.3 × 10^8^ colony‐forming units (CFU) g^−1^] than that on healthy carrots (1.8 × 10^7^ CFU g^−1^) (Fig. 3). The number of bacteria on brownish carrots increased to 2.7 × 10^9^ CFU g^−1^ (Fig. 3).

### CO_2_‐rich environment modifies carrot microbiome

To determine the effect of high ambient CO_2_ on the carrot microbiome, the microbial population of carrots exposed to a 98% CO_2_ environment was analysed in four phenological stages: ‘healthy’ (time 0), ‘pre‐oozing’ (a day before visible oozing), ‘oozing’ and ‘brownish’ (24‐h ventilation after oozing).

**Figure 4:**
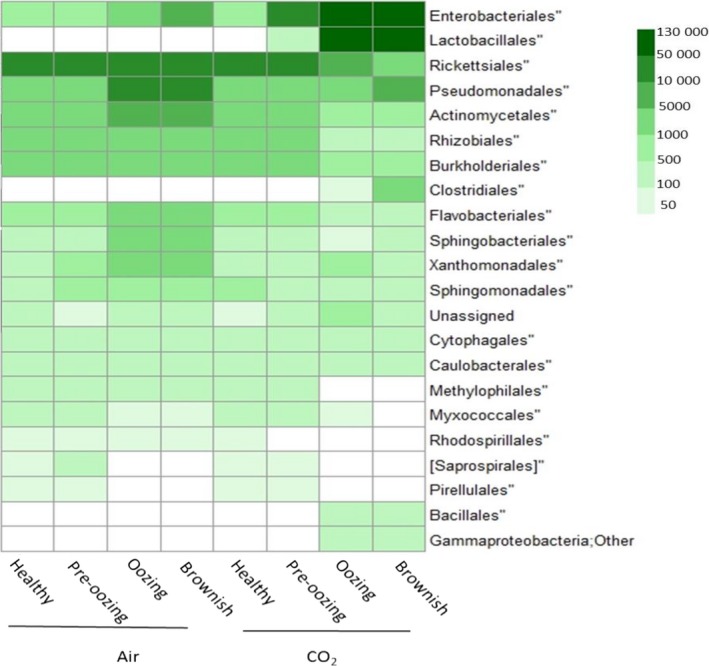
Heat map of average normalized counts suggesting that CO_2_ environment changes the microbiome on the carrot surface. The microbial population of carrots exposed to air or 98% CO_2_ environment was analysed at four phenological stages: healthy (time 0), pre‐oozing (a day before visible oozing), oozing and brownish (24‐h ventilation after oozing). The scale of the heat map represents abundance in major bacterial population distribution at the order level (L4). The abundance of the different bacteria at the order level was normalized using the trimmed mean of M‐values normalization method (TMM), and then, all replicates were averaged to create the mean normalized level of the bacterial population.

A heat map was used to show the relative normalized abundance within each sample of the most abundant microbial orders (Fig. 4). Carrots incubated in air were dominated by *Rickettsiales* and a lower level of *Pseudomonadales*,* Actinomycetales*,* Rhizobiales* and *Burkholderiales* in the healthy and pre‐oozing stages (Fig. 4). In later stages of incubation, low severity of oozing and brownish increased levels of *Enterobacteriales, Pseudomonadales*,* Actinomycetales*,* Flavobacteriales*,* Sphingobacteriales* and *Xanthomonadales* (Fig. 4). In contrast, carrots incubated in the CO_2_‐rich environment were dominated by *Lactobacillales* and *Enterobacteriales*, mainly during oozing and brownish stages (Fig. 4).

The alpha diversity value, representing mean species diversity in each treatment, reflected minor differences for the air‐exposed samples and significant differences for the CO_2_‐exposed samples over time (Table 1). During the oozing process occurring in the CO_2_‐rich environment, the number of operational taxonomic units (OTUs) decreased (Table 1). The air samples and the pre‐oozing CO_2_ samples showed higher species richness (OTUs) than the oozing and brownish CO_2_ samples (Table 1). The former group was more diverse as well, according to its higher Shannon diversity index compared to the CO_2_‐oozing and CO_2_‐brownish samples (Table 1). Similarly, the single rarefaction curves (not shown) computed for each sample by Chao1 richness estimator showed similar trends per sample (Table 1).

**Table 1:** Average alpha diversity for carrot samples exposed to air or CO_2_

	OTUs (97%)		Coverage		Shannon		Chao	
	Average	STDEV	Average	STDEV	Average	STDEV	Average	STDEV
Air								
Healthy	4847 ab^a^	652.02	0.96	0.008	4.490 ab	0.57	11 034.146 a	1270.90
Pre‐oozing	5030 a	699.88	0.96	0.008	4.3163 a	0.59	11 306.765 a	1470.05
Oozing	6534 a	382.76	0.97	0.003	5.2607 ab	0.52	13 869.607 a	765.38
Brownish	6479 a	810.61	0.97	0.002	4.884 ab	0.44	13 642.255 a	1488.41
CO_2_								
Healthy	5070 a	358.18	0.96	0.004	4.773 ab	0.56	11 204.286 a	732.59
Pre‐oozing	5643 a	677.81	0.96	0.004	4.720 ab	0.66	12 686.648 a	1618.00
Oozing	4199 c	253.24	0.98	0.002	4.109 b	0.18	7517.282 b	554.90
Brownish	4326 bc	385.80	0.98	0.002	4.154 ab	0.25	7765.346 b	855.83

a Different letters indicate significant difference based on one way ANOVA (*P* < 0.05), followed by Tukey HSD test.

**Figure 5:**
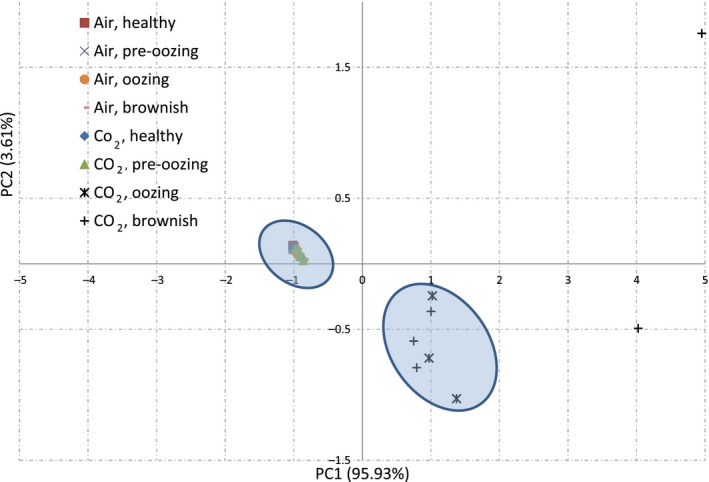
PCA biplot calculated with factomineR. The microbial population of carrots exposed to air or 98% CO_2_ was analysed at four phenological stages: healthy (time 0), pre‐oozing (a day before visible oozing), oozing and brownish (24‐h ventilation after oozing).

The bacterial community compositions differed considerably between the air‐ and CO_2_‐stored carrots, when carrot oozing began. A sharp difference in community structure between the CO_2_‐oozing and brownish samples and all other samples are clearly illustrated by the PCA biplot and the topology of the weighted UniFrac UPGMA tree (Figs 5 and S1 respectively). All of the air samples, and the CO_2_‐healthy and CO_2_‐pre‐oozing samples, are clustered tightly in the biplot graph, indicating a similar community composition in these samples. The CO_2_‐oozing and CO_2_‐brownish samples, on the other hand, are clustered separately, albeit not as tightly as the other samples (Fig. 5). The UniFrac tree supports the PCA graph, with the CO_2_‐oozing and CO_2_‐brownish samples forming one cluster, and the other samples forming a separate cluster, indicating a distinct difference between the bacterial community compositions of healthy and oozing carrots (Fig. S1).

### Isolation of microorganisms from oozing carrots

Cultivation of oozing and brownish carrot samples resulted in the appearance of three prevalent bacterial morphotypes on the agar media, which differed in colour and size. To determine whether each of these three apparent morphotypes make up a homogeneous population, five colonies of each morphotype were further isolated to pure cultures and subjected to molecular identification by 16S rRNA gene sequence analysis. The three morphotypes were identified as *Pantoea agglomerans* (99% similarity, GenBank accession number NR041978), *Rahnella aquatilis* (99% similarity, GenBank accession number NR074921) and *Leuconostoc mesenteroides* (100% similarity, GenBank accession number NR074957). *P. agglomerans* and *R. aquatilis* are Gram‐negative bacteria of the family *Enterobacteriaceae*, while *L. mesenteroides* is a Gram‐positive lactic acid bacterium of the family *Leuconostocaceae* (Krieg and Holt, 1994). These findings are in agreement with the microbiome analysis as *L. mesenteroides* belongs to the order *Lactobacillales*, while *P. agglomerans* and *R. aquatilis* are members of the order *Enterobacteriales*.

### Effect of CO_2_ on growth of *P. agglomerans, R. aquatilis* and *L. mesenteroides*

The presence of abundant bacterial populations of *P. agglomerans, R. aquatilis* and *L. mesenteroides* on oozing and brownish carrot tissues under high levels of CO_2_ suggested a growth advantage for these bacteria in the CO_2_‐rich atmosphere. To test this, serial dilutions of the three bacteria were plated on carrot agar medium and incubated for 5 days at 20°C in a 98% CO_2_ atmosphere. *L. mesenteroides* showed rich growth, while *R. aquatilis* grew poorly and *P. agglomerans* did not grow at all under these conditions. All three strains showed comparable growth levels upon ventilation in ambient air for 3 days (Table 2).

**Table 2:** Growth of bacterial isolates derived from oozing and brownish carrot peels

Most closely related hit in GenBank	% Identity (> 500 bp)	Bacterial taxa (class, family)	Level of growth: CO_2_ incubation	Level of growth: air incubation (after CO_2_)
*Pantoea agglomerans* (NR041978)	99	*Gammaproteobacteria, Enterobacteriaceae*	−	+++
*Rahnella aquatilis* (NR074921)	99	*Gammaproteobacteria, Enterobacteriaceae*	+	+++
*Leuconostoc mesenteroides* (NR0974957)	100	*Bacilli, Leuconostocaceae*	+++	+++

(−) no growth; (+) poor growth of few colonies; (+++) colonies cover of the entire Petri dish.

### *L. mesenteroides* induces oozing on carrot discs

**Figure 6:**
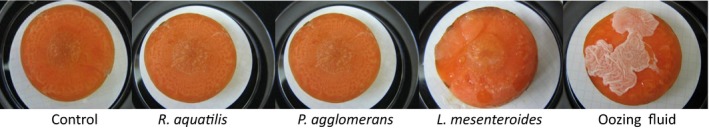
Representative images showing inoculated carrot discs. A.Control, no bacteria. B. *R. aquatilis*. C. *P. agglomerans*. D. *L. mesenteroides*. E. Oozing fluid, after 5 days at 20°C.

As only *L. mesenteroides* grew in the CO_2_‐rich atmosphere, this bacterium might be the causative agent of the oozing symptoms. To examine the role of each strain in oozing, Koch's postulates were tested by inoculating carrot discs with each of the three strains, as well as with fluid from the exudate, followed by incubation in a controlled 98% CO_2_ atmosphere or ambient air for 5 days. Only *L. mesenteroides* and the carrot exudate, and not *P. agglomerans* or *R. aquatilis*, induced oozing symptoms on the carrot discs in the CO_2_ atmosphere (Fig. 6). None of the bacterial strains, nor the fluid exudate, were able to induce oozing symptoms following incubation in ambient air. R‐2A Broth, used as a control, did not induce any symptoms either in both of the conditions (Fig. 6). To confirm that *L. mesenteroides* is indeed the causative agent of the oozing, Koch's postulates were completed by reisolating *L. mesenteroides* from the oozing fluid that developed on the *L. mesenteroides*‐inoculated carrot discs.

These findings supported the notion that high CO_2_ level supports the growth of the anaerobic bacterium *L. mesenteroides*. However, it could be that the high CO_2_ level stressed the carrot tissue, making it more susceptible to bacterial proliferation, regardless of atmospheric composition. To test this notion, carrot discs were pre‐exposed to 98% CO_2_ from 5 h to 5 days before bacterial inoculation as indicated above. Following inoculation, the discs were stored in ambient air for an additional 5 days. None of the discs displayed any oozing symptoms, suggesting that previous CO_2_ stress alone is not sufficient to support oozing symptoms under ambient air in the presence of each of the three species.

## Discussion

### Non‐ventilated conditions modify microbiome composition

In the current study, we developed a small‐scale model system that simulates the environmental conditions during long‐term storage of commercially brushed carrots under non‐ventilated conditions. During storage of carrots, which are harvested at full metabolic activity (Godfrey and Marshall, 2002), non‐ventilated conditions allow the build‐up of CO_2_ concentration, while O_2_ concentration gradually decreases (Fig. 1). These environmental changes can induce the development of oozing, previously reported in stored ‘ready‐to‐use’ grated carrots packed in polymeric films, where the combination of high CO_2_ and low O_2_ concentrations was shown to trigger spoilage (Carlin *et al*., 1990a,b). Spoilage because of growth of lactic acid bacteria (LAB) is well documented in cold‐stored, vacuum‐packaged or modified atmosphere‐packaged meat, poultry and processed meat products (Vihavainen *et al*., 2008). In these products, LAB spoilage is often characterized by deteriorations in odour, flavour, colour or appearance (reviewed by Remenant *et al*., 2015). However, it is important to mention that high CO_2_ can have a dual effect on different microbial populations. Modified atmosphere may significantly inhibit spoilage organisms as well as desirable microflora in post‐harvest produce, due to the non‐selective antimicrobial effect of CO_2_ (Farber *et al*., 2003; Caleb *et al*., 2013).

The main changes observed in the carrot microbiome during storage in the non‐ventilated environment were increased abundance of *Enterobacteriales* and *Lactobacillales* and decreased abundance of *Rickettsiales*. These changes are likely triggered by alterations in the atmosphere's gaseous composition (increased CO_2_, low O_2_). Elevated CO_2_ levels have been previously reported to affect the composition of microbial communities on other agricultural produce (Gill, 1996; de Oliveira *et al*., 2010; Lo *et al*., 2016). The antimicrobial effect of CO_2_ has been reported to affect bacterial and viral population density, and gene expression of some foodborne‐pathogen toxins (Francis *et al*., 1999; Bidawid *et al*., 2000; Artin *et al*., 2008). Whereas aerobic bacteria such as pseudomonads are inhibited by moderate to high levels of CO_2_, other microorganisms, such as the facultative anaerobic LAB and yeast, can be stimulated under these conditions (Enfors and Molin, 1980; Amanatidou *et al*., 1999; Soliva‐Fortuny and Martín‐Belloso, 2003; Oliveira *et al*., 2010).

*Rickettsiales* are small aerobic bacteria in the class *Alphaproteobacteria*. They have a diverse host range as endosymbionts, including arthropods, vertebrates and plants (Weinert *et al*., 2009). *Rickettsia*‐related organisms were found in diseased plants from various species (including in carrots) although it is not clear whether they are the actual disease agents (Franova *et al*., 2000; Streten *et al*., 2005; Luis‐Pantoja *et al*., 2015). Reduction in the abundance of *Rickettsiales* in a CO_2_‐rich atmosphere suggests that they are sensitive to high CO_2_ concentration, similar to other Gram‐negative bacteria.

Storage of carrots in a non‐ventilated or CO_2_‐rich atmosphere resulted in decreased population richness and diversity (Figs 4, 5, 6). This might be associated with growth limitation in CO_2_‐rich atmosphere, as reported, for example, during storage of meat under modified atmosphere (Stoops *et al*., 2015). Similarly, decreased species richness and diversity have also been reported during storage of spinach and fresh‐cut lettuce under modified atmosphere (Lopez‐Velasco *et al*., 2011; Di Carli *et al*., 2016).

Expression of antibacterial compounds, known to be secreted by many LAB species (Cotter *et al*., 2013), may also have contributed to the decreased species richness and diversity. Other causes for the population changes might include competition for carrot‐derived nutrients and adaptation to the changing environment.

### *L. mesenteroides* is the causative agent of oozing

Induction of oozing was associated with increased bacterial counts on the root surface (Fig. 3), and the abundance of three dominant species, *P. agglomerans, R. aquatilis* and *L. mesenteroides*, all of which could potentially be involved in carrot spoilage. However, only *L. mesenteroides* induced oozing on infected carrot discs under high CO_2_ atmosphere (Fig. 6). Nevertheless, inoculation of carrot discs with exudate derived from oozing whole roots resulted in more extensive symptoms than that induced by *L. mesenteroides* alone (Fig. 6). This might indicate the involvement of additional microorganisms in the soft rot of naturally infected roots, which were absent in our model of sterilized carrot discs. Indeed, rotten vegetables are frequently colonized by secondary bacterial or fungal pathogens which may even mask the primary spoilage pathogen (Godfrey and Marshall, 2002).

The other two species isolated from the oozing carrots are also considered to be natural soil and plant flora. *P. agglomerans* is abundant in soil and plants (Monier and Lindow, 2005; Son *et al*., 2006) and colonizes numerous types of vegetables and fruits, including sprouts, spinach, lettuce, pepper, strawberries (Leff and Fierer, 2013), potato tubers (Sturz *et al*., 1999) and onion (Dutta *et al*., 2014). Similarly, *R. aquatilis* is a common soil and plant inhabitant (Kim *et al*., 1997; Calvo *et al*., 2007; Chen *et al*., 2007). Hausdorf *et al*. (2011) found both *P. agglomerans* and *R. aquatilis* in wash water from an industrial carrot washing and packing plant. It is possible that these bacteria were merely secondary colonizers that flourished in the nutrient‐rich exudate generated by *L. mesenteroides*.

*Leuconostoc mesenteroides* is a lactic acid bacterium (LAB) associated with the fermentation of vegetables and other products (Hemme and Foucaud‐Scheunemann, 2004; Wouters *et al*., 2013). It has been isolated from lettuce (Leff and Fierer, 2013) and fresh‐cut celery (Robbs *et al*., 1996), as well as from grated carrots (Carlin *et al*., 1990a) and spoiled vacuum‐packaged vegetable sausages (Vihavainen *et al*., 2008). Known causative agents of soft rot in stored carrot include *Pseudomonas viridiflava* and *Pseudomonas marginalis* (Godfrey and Marshall, 2002; Almeida *et al*., 2013), *Pectobacterium (Erwinia) carotovorum* and *Pectobacterium (Erwinia) chrysanthemi* (Towner and Beraha, 1976), *Rhizoctonia carotae* (Geeson *et al*., 1988) and *Sclerotinia sclerotiorum* (Liew and Prange, 1994).

To the best of our knowledge, our study is the first to implicate *L. mesenteroides* in oozing of partially processed carrot roots during storage under non‐ventilated conditions, although its involvement in soft rot of ‘ready‐to‐use’ grated carrots packaged in polymeric films is known (Carlin *et al*., 1990a). It is likely that *L. mesenteroides* is an innocuous inhabitant of the carrot root surface, which became an opportunistic pathogen due to the commercial brushing process followed by high respiration and low ventilation of the transported carrot. Brushing or grating of the root surface results in loss of the epidermal tissue, which exposes the inner nutrient‐rich tissues to the intrinsic microbial flora.

High levels of CO_2_ can inactivate certain microorganisms, including bacteria, yeast, fungi and their spores (reviewd by Damar and Balaban, 2006). It is likely that nutrients derived from the bruised tissue in combination with high CO_2_ atmosphere provided a selective growth advantage to *L. mesenteroides*. Once the chambers are opened and ventilated, the ambient air together with nutrients that are available in the exudates may provide conditions favouring the growth of other species present in the same ecological niche, such as *P. agglomerans* and *R. aquatilis*.

None of the three predominant species were able to cause oozing symptoms on carrot discs under ambient air conditions (Fig. 6). While *L. mesenteroides* presented rich growth on carrot agar medium under high CO_2_ and ambient air environments (Table 2), oozing symptoms only occurred on carrot discs with high CO_2_ levels (Fig. 6). Thus, the pathogen grows and initiates oozing mainly in a CO_2_‐rich environment. It can be assumed that high CO_2_ concentration induces virulence genes required for the production and secretion of exudate by *L. mesenteroides*, as other studies have suggested that CO_2_ regulates the expression of virulence factors involved in bacterial pathogenesis (Finlay and Falkow, 1989; Miller *et al*., 1989). Similarly, the aggressiveness of the plant pathogens *Colletotrichum gloeosporioides* and *Erysiphe cichoracearum* was found to increase in the presence of elevated CO_2_ concentrations (Chakraborty and Datta, 2003; Lake and Wade, 2009). Interestingly, a recent study has indicated increased quorum‐sensing‐mediated virulence in soft rot caused by *Pectobacterium carotovorum* under elevated temperature and CO_2_ levels (Das and Chaudhary, 2014). Transcriptome profiling of *L. mesenteroides* under low‐ and high‐CO_2_ atmospheres may shed light on the genes and mechanisms responsible for the oozing phenomenon in *L. mesenteroides*‐infected carrot under non‐ventilated conditions.

In summary, this study pinpoints a case in which consumer preference for aesthetic carrot appearance combined with technological advances (washing and mechanical brushing) and trade globalization have led to the emergence of an economically important disease by a seemingly opportunistic pathogen that until now had only rarely been associated with carrot spoilage. Characterization of the conditions associated with the shipping of stored carrots and better control of the ventilation conditions will probably open the way to developing intervening means to prevent spoilage of shipments, and the associated financial losses.

## Experimental procedures

### Plant material, processing and storage conditions

Carrot roots (*Daucus carota* L.) cv. Nairobi were grown in the northern Negev (in southern Israel). They were harvested in the spring (April), washed in water to remove soil residue and brushed in a commercial machine using spiral‐wound coil brushes made of 0.5‐mm polyester filaments, which resulted in peeling the epidermis from the roots. After brushing, carrots were hydro‐cooled in a 4°C water bath, packed in commercial 12‐kg polyethylene bags and stored at 1°C for 2 months until further analysis.

### Storage experiments and analysis of volatiles

Glass chambers (2 l) equipped with a rubber septum were washed and disinfected by spraying with 70% ethanol for 1 min and wiping with a paper towel. The chambers were left open for 5 min so that the ethanol residue would evaporate. Brushed carrots (9–12) were placed in each chamber, occupying approximately 75% of its volume. Chambers were hermetically sealed or air‐ventilated by keeping the upper septum open. To enhance spoilage symptoms, the chambers were kept in the dark at 20°C for a further 15–17 days. The chambers were then opened and exposed to ambient atmosphere for up to 48 h. Visual symptoms were monitored daily throughout the storage period.

Volatiles were sampled from the headspace of each chamber by inserting a syringe through the septum. CO_2_ levels were determined by gas chromatography using a thermal conductivity detector (Series 580; GOW‐MAC Instrument Co, Bethlehem, PA, USA). Oxygen levels were determined by Oxybaby gas analyser (model 6; WITT‐Gasetechnik GmbH and Co KG, Witten, Germany). Acetaldehyde and ethanol levels were determined with a Varian 3300 gas chromatograph (Hewlett‐Packard, Bloomington, IL, USA). Ethylene levels were determined with a Varian 3300 gas chromatograph (Walnut Creek, CA, USA).

In some experiments, the carrot‐containing chambers were flushed daily for 7 days with CO_2_, N_2_ or air to maintain a controlled atmosphere. The level of the gas in each chamber was confirmed daily, immediately after flushing, as described above. The gas levels were as follows: 98% CO_2_, 98% N_2_, and for air: 78.9% N_2_, 20.8% O_2_ and 0.3% CO_2_.

### Microbial analysis

During storage in the non‐ventilated chambers, an exudate appeared on the carrot surface, a phenomenon known as oozing. Carrots that changed their colour to brown, 24 h after opening and ventilating the chamber, were termed ‘brownish’. Peels of ‘healthy’, ‘oozing’ and ‘brownish’ carrots (five carrots from each chamber) were individually sampled using a sterile kitchen peeler and were transferred into sterile 15‐ml polypropylene tubes. Peel (5 g) was ground in a laboratory blender in 50 ml of saline in a sterile cup. Microbial enumeration was performed by standard serial dilutions and plating in triplicate on three media: (i) PCA (Difco, Sparks, MD, USA); (ii) carrot agar, a medium which was prepared by mincing four carrots in 1 l distilled water supplemented with 1.5% Bacto Agar (Difco); (iii) PDA (Difco) with addition of 0.25 mg ml^−1^ chloramphenicol. All plates were incubated at 28°C for 2 or 7 days (for bacteria and fungi respectively), and the number of CFU was determined. The prevalent bacterial colonies, based on colony phenotype, were isolated and subcultured on PCA plates to obtain pure cultures. Isolated colonies from pure cultures were kept in 30% glycerol stocks and maintained at −80°C.

### Microbiome sample collection

Ten 2‐l glass chambers were filled with carrots (cv. Nairobi). Five chambers were sealed and flushed daily with 100% CO_2_ and the other five were left open (ventilated). To enhance symptom appearance, the chambers were kept in the dark at 20°C, as above, for 21 days. Microbial samples were taken from the carrot surface at four time points by swabbing. The first sample (‘healthy’) was taken at the beginning of the experiment (time 0). A second sample was taken after 18 days of incubation (‘pre‐oozing’), and a third sample was taken after 20 days (‘oozing’). After the third sampling, all of the chambers were opened and exposed to ambient air. The final sample was taken 24 h later, on day 21 (‘brownish’). Each sample included five repeats of 1.0‐ml sterilized double‐distilled water (sddw) used to resuspend the swab from one carrot. The samples were stored immediately after sampling at −80°C until further use.

DNA was extracted from the different samples using Exgene Soil DNA Isolation kit (GeneAll Biotechnology, Seoul, Korea). DNA concentration was determined in a Nanodrop ND1000 spectrophotometer (NanoDrop Technologies, Wilmington, DE, USA). The DNA samples were stored at −80°C until use.

### 16S rRNA sequencing analysis

Amplicons for barcoded paired‐end Illumina Miseq sequencing (Illumina, San Diego, CA, USA) were generated using the primer set 515F (5′‐GTGCCAGCMGCCGCGGTAA‐3′) and 806R (5′‐GGACTACVSGGGTATCTAAT‐3′) that amplifies the bacterial V4 hypervariable region as described previously (Caporaso *et al*., 2012). Overall, 4 765 126 amplicons were sequenced. All sequence pre‐processing, analysis of OTUs and classifications were performed using QIIME version 1.9.0 (Caporaso *et al*., 2010a). In the first step, PEAR software version 0.9.6 (Zhang *et al*., 2014) was used to merge the fastq files, and then, quality and length trimming was performed using CLC Bio software (http://www.clcbio.com/). Chimeras were removed using USEARCH (Edgar, 2010), and finally, taxonomic assignment was performed with the script assign_taxonomy.py against Greengenes QIIME datafiles version 13_8 (DeSantis *et al*., 2006). OTUs were generated using a 97% sequence‐identity threshold. OTUs with less than two observations, and samples with fewer than 150 observations were filtered from the OTU table. Phylogenetic analysis was performed by aligning the representative sequences with PyNAST (Caporaso *et al*., 2010b) and then creating the tree using the FASTTREE algorithm (Price *et al*., 2009). Community profiles were compared using the weighted UniFrac metric (Hamady *et al*., 2010), and a UPGMA tree was constructed with QIIME based on the UniFrac metric.

### Molecular identification of isolated bacteria

Identification of bacterial isolates was performed by sequencing of the 16S rRNA gene. Briefly, five colonies of each prevalent isolate were picked with a sterile bacteriological needle and resuspended separately in 10 μl of filtered sddw. The suspension was heated at 95°C for 5 min and then centrifuged for 10 min at 13 800 *g* to remove intact bacterial cells and large debris. The supernatant containing the DNA was used as a template for PCR amplification using the universal bacterial primers 8–27: 5′‐AGAGTTTGATCCTGGCTCAG‐3′ and 1492: 5′‐GGTTACCTTGTTACGACT‐3′ (Lampert *et al*., 2006) as follows: 5 min of denaturation at 94°C; 30 cycles of 1 min at 94°C, 1 min at 52°C and 1 min at 72°C; termination at 72°C for 10 min. The amplified DNA fragments were purified from an agarose gel using the Zymoclean Gel DNA Recovery kit (The Epigenetics Company; Zymo Research Corp., Irvine, CA, USA) and sent for sequencing (Hylabs Ltd., Rehovot, Israel). Sequence data were compared to the 16S rRNA gene database in the National Center for Biotechnology Information (NCBI) GenBank database, using the BLAST program (Altschul *et al*., 1990).

### Laboratory models for carrot inoculation

Carrots were surface‐sterilized by dipping in 1% sodium hypochlorite solution (Bio‐Lab, Jerusalem, Israel) for 1 min and immediately washed by dipping in sddw for 30 s. Surface‐sterilized carrots were peeled or horizontally cut by sterile scalpel blade to sample the cortex or central core tissues respectively. The presence of microorganisms in these tissues was determined as described above for the peels.

Carrot discs (ca. 5 mm width and 30–35 mm diameter) were placed in a sterile Petri dish containing a 0.22‐μm filter paper (47 mm; Millipore, Merck) soaked with 600 μl sddw. A 50‐μl aliquot of either pure culture of the tested strain (10^8^ CFU ml^−1^) in R2 Broth (Hi Media Laboratories Pvt., Mumbai, Maharashtra, India) grown at 28°C for 24 h, or carrot exudate collected from oozing carrots and kept at 4°C for 24 h, was placed at the centre of each disc. The Petri dishes were placed in the above‐described glass chambers, which were flushed daily with 98% CO_2_ or ambient air, and kept at 20°C for up to 5 days, as described above for intact carrots.

In another set of experiments, sterile carrot discs were pre‐exposed to 98% CO_2_ for various periods of time ranging from 5 h to 1–5 days before inoculation with bacteria or with exudate fluid. The inoculated discs were left in ambient air for another 5 days at 20°C. All of the carrots discs were examined daily for oozing and browning symptoms.

## Conflict of interest

None declared.

## Supporting information


**Fig. S1.** Unweighted UniFrac distance Jackknife dendrogram of bacterial communities.
