# Supplementary figures and images for: Emergence of Leuconostoc mesenteroides as a causative agent of oozing in carrots stored under non‐ventilated conditions
Source: Microb Biotechnol. 2017 Aug 22;10(6):1677–89. doi: 10.1111/1751-7915.12753 (PMC5658626; doi:10.1111/1751-7915.12753)

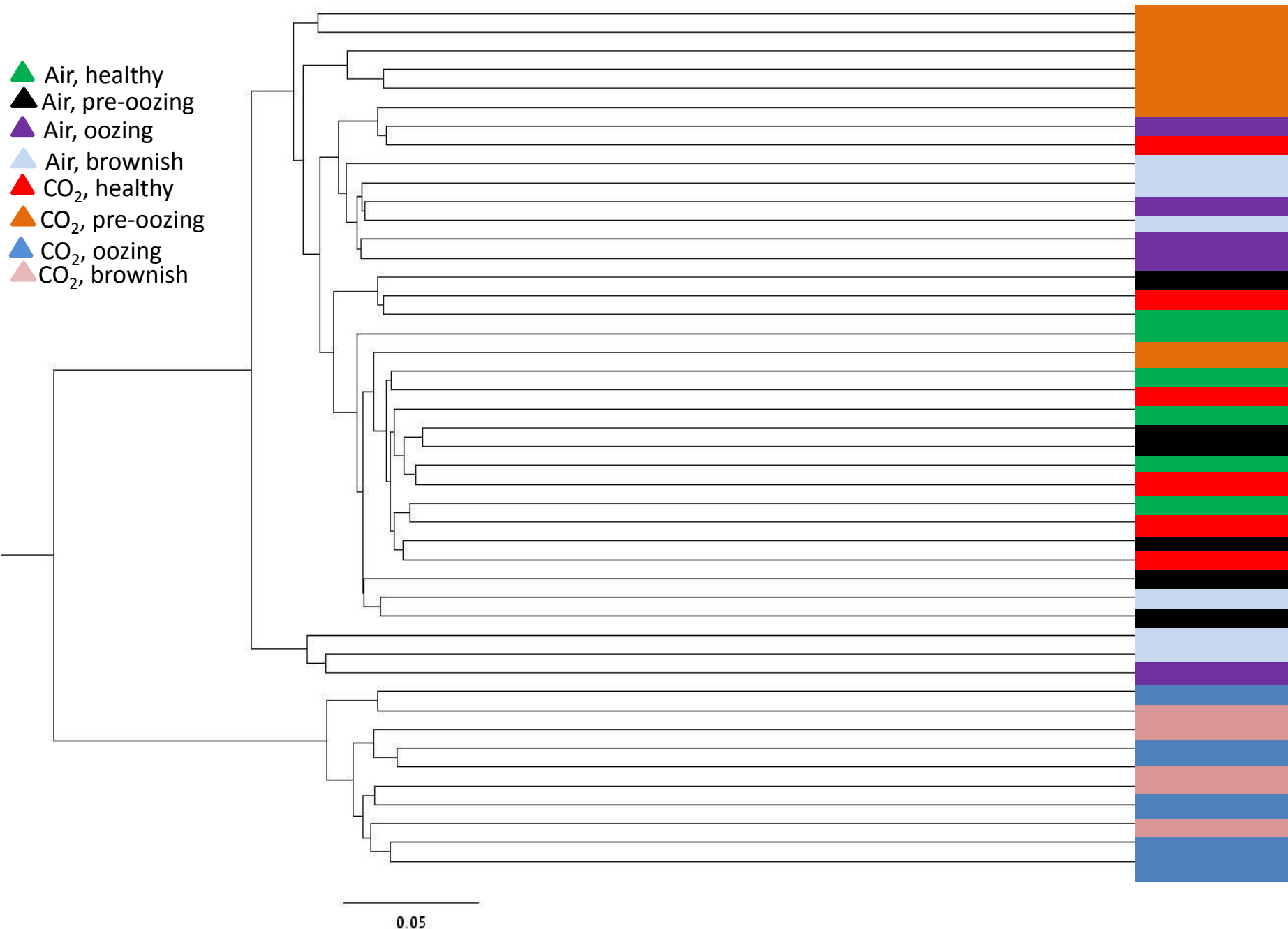

Supplement: Supplementary file 1 — Fig. S1. Unweighted UniFrac distance Jackknife dendrogram of bacterial communities. [file MBT2-10-1677-s001.pdf]
